# Enhancing Oral Absorption of Quercetin Through Multifactorial Synergies in Crystal Dispersion Systems

**DOI:** 10.3390/molecules30112390

**Published:** 2025-05-30

**Authors:** Yao Liu, Qiuli Yan, Chunhui Hu

**Affiliations:** Department of Pharmacy, Qinghai University, Xining 810001, China; zhangyong20100507@163.com (Y.L.); chunhuihu@hotmail.com (Q.Y.)

**Keywords:** quercetin, crystalline solid dispersion, dissolution rate, oral absorption, permeability

## Abstract

This study aims to enhance the dissolution rate and oral absorption of quercetin (QUR) by formulating quercetin crystalline solid dispersion (QUR-CSD). Quercetin, as a natural antioxidant, can effectively neutralize free radicals, reduce inflammatory responses, help lower the risk of cardiovascular diseases and certain cancers, and support the function of the immune system. CSDs underwent characterization through powder X-ray diffraction and scanning electron microscopy, and dissolution rates were evaluated in vitro. Oral absorption assessment was conducted using SD rats, while Caco-2 monolayer cell transmembrane (CMCT) and single pass intestinal perfusion (SPIP) were performed to assess the permeability of CSDs. QUR within the CSDs exhibited hydrogen bond interactions with P188 and PEG, displaying stronger interaction parameters (χ) of –4.0 and –6.1, respectively. The crystalline domain of QUR within Poloxamer 188 (P188) was smaller than within polyethylene glycol 8000 (PEG8000). CSDs improved the dissolution rate of QUR, with the P188-CSD slightly outperforming the PEG8000-CSD due to P188’s ability to enhance drug wettability and solubility and to maintain supersaturation. Pharmacokinetic results revealed a 3.5-fold and 25-fold increase in oral absorption for P188-CSD and PEG8000-CSD, respectively, compared to QUR. CMCT and SPIP indicated superior permeability for PEG8000-CSD, potentially attributed to caveolin-mediated PEG transmembrane transport. QUR-CSD significantly enhanced oral absorption, with PEG8000-CSD demonstrating superior efficacy. This improvement was attributed to various factors, including crystalline size reduction, drug wettability enhancement, maintenance of supersaturation by polymers, and caveolin-mediated transmembrane transport.

## 1. Introduction

Quercetin (QUR), or 3,3′,4′,5,7-pentahydroxyflavone, is one of the most common plant-derived flavonoids and is widely present in the normal human diet; it is present in high levels in foods such as onions, shallots, asparagus, apples, and wine [1,2,3]. In the United States and China, the average dietary intake of QUR is estimated to be 20 mg/day. Epidemiological studies have identified the potential of QUR for the prevention of cardiovascular disease and diabetes, as well as studying its anti-cancer, anti-inflammatory, and antiviral effects. However, despite the extensive health benefits of QUR, its low water solubility (a mere 0.00215 g/L at 25 °C) and limited gastrointestinal permeability severely affect the absorption of QUR [4,5]. Therefore, there is an urgent need to design formulations for optimal QUR absorption and total absorption to better realize its multiple health benefits. Numerous techniques have been explored to enhance its solubility, including nanoparticles [6], micelles [7], nanoemulsions [8,9], and solid dispersions [5,10]. However, many of these formulations result in amorphous solid dispersions, which, while improving solubility by disrupting the drug crystalline lattice, may exhibit thermodynamic instability, making them unsuitable for long-term storage.

Contrary to these unstable high-energy states, author focus has been on developing a thermodynamically stable solubilization system known as crystalline solid dispersion (CSD), wherein drugs exist in a microcrystalline state. Investigations have revealed that reducing drug crystalline size within the polymer matrix plays a pivotal role in enhancing drug dissolution rates, as per the Noyes-Whitney dissolution rate equation [11]. Our previous studies have demonstrated that the drug crystalline size in CSD decreases in the polymer [12,13]. As observed from the microstructure, the regulatory mechanism of polymers mainly includes steric hindrance, glass transition temperature [14], and drug–polymer interaction [15,16].

Poloxamer (or Pluronic) and polyethylene glycol (PEG) polymers have emerged as common choices for preparing CSD due to their distinctive properties. Poloxamer, a triblock (PEO-PPO-PEO) copolymer, and PEG, a homopolymer, exhibit varying effects on the microstructure [17,18], in vitro dissolution, and in vivo pharmacokinetics of CSD owing to the differences in crystallization kinetics and microstructure.

In addition to solubility and permeability, drug absorption hinges on the influence of efflux proteins. Polymers can directly impact the function of efflux proteins. For instance, Poloxamer 188 (P188) and PEG8000 have been shown to inhibit P-glycoprotein (P-gp) efflux or enhance cell membrane permeability. PEG additionally facilitates caveolin-mediated transmembrane transport [19], underscoring its pivotal role in enhancing drug absorption.

In this study, we employed QUR as a model drug and utilized P188 and PEG8000 as carriers to prepare two CSDs aimed at enhancing QUR’s dissolution rate and oral absorption. Comprehensive characterization of CSD formulations encompassed hydrogen nuclear magnetic resonance spectroscopy (^1^H-NMR) to examine intermolecular interactions, powder X-ray diffraction (PXRD), laser particle size analyzer (PSA), and scanning electron microscopy (SEM) to evaluate micromorphology and crystalline size. The in vitro dissolution rate of CSD formulations was assessed through intrinsic dissolution rate and pH conversion two-step dissolution methods. Pharmacokinetic studies conducted in SD rats validated the enhanced oral absorption conferred by CSD formulations. Finally, in vitro cell permeability assays involving Caco-2 monolayer cells and in vivo single pass intestinal perfusion experiments were conducted to further elucidate drug absorption. This comprehensive study aimed to characterize and compare two CSD formulations. The influencing factors of CSD constructed with different polymer materials increasing the oral bioavailability of quercetin were explained from multiple dimensions in basic theory. In practical applications, the present research can provide new and feasible solubilization methods for the formulation design of such poorly soluble drugs.

## 2. Results and Discussion

### 2.1. Determination of Solubility Parameter and Interaction Parameter χ Value

The compatibility of drugs and polymers can be predicted by calculating the solubility parameter value (Δδt). Better compatibility indicates stronger interactions. Generally, if Δδt (MPa^0.5^) < 7, a drug exhibits good compatibility with the polymer; smaller values indicate better compatibility. Conversely, Δδt (MPa^0.5^) > 10 suggests poor compatibility between the drug and the polymer [20]. In this study, the Δδt between QUR and P188 or PEG8000 were 2.62 (J·cm^−3^)^1/2^ and 2.37 (J·cm^−3^)^1/2^, respectively (Table 1), indicating good compatibility between QUR and both polymers.

To further assess drug–polymer miscibility, we calculated the χ value between the drug and the polymer by measuring the equilibrium temperature of the drug–polymer mixture using DSC. According to Flory–Huggins parameter theory, drug–polymer interaction is key to evaluating system uniformity. A χ value < 0 indicates good miscibility, while χ > 0 suggests poor miscibility and a tendency for phase separation, leading to system instability [21]. The results showed χ_QUR-P188_ and χ_QUR-PEG8000_ were –4.0 and –6.1, respectively, consistent with the solubility parameter predictions. This suggests that the QUR-PEG8000 system was more uniform, stable, and miscible than the QUR-P188 system.

### 2.2. Intermolecular Interactions

**Figure 1:**
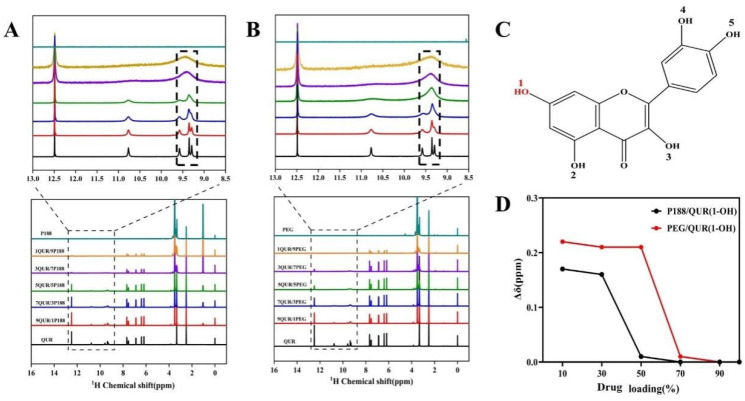
^1^H-NMR spectra of QUR/P188-CSD and QUR/PEG8000-CSD. (**A**) ^1^H-NMR spectrum of QUR/P188-CSD; (**B**) ^1^H-NMR spectrum of QUR/PEG8000-CSD; (**C**) Molecular structure of quercetin; (**D**) Comparison of the 1-OH shift of quercetin.

In this study, NMR, a highly sensitive technical tool, was employed to elucidate the complex interactions between drug and polymer molecules [22]. NMR analysis focused on the displacement (σ) changes of 1-OH in the QUR structure upon interaction with the polymer (Figure 1A,B). The authors found that the 1-OH in QUR exhibited more significant displacement changes compared to the 2,3,4,5-OH (Figure 1C). In QUR/P188-CSD, the σ of the 1-OH changed from 9.58 ppm to 9.41 ppm, with a difference (Δσ) of 0.17 ppm. In QUR/PEG8000-CSD, the Δσ of the 1-OH was 0.22 ppm (Figure 1D). The Δσ suggested that QUR formed stronger hydrogen bonding interactions with PEG8000 compared to P188 [21]. However, the 2,3-OH tended to form intramolecular hydrogen bonds with the carbonyl group within the molecule, thereby weakening the intermolecular hydrogen bond interactions. Consequently, the 1-OH had stronger hydrogen bonding activity, making it the primary site for forming intermolecular hydrogen bonds. Therefore, 1-OH was the main binding site for forming hydrogen bonds with the outside world [23]. QUR exhibited strong hydrogen bond interactions with both P188 and PEG8000 polymers. However, the interaction strength with PEG8000 was greater than that with P188.

### 2.3. Crystallization Kinetics and Crystalline Size

**Figure 2:**
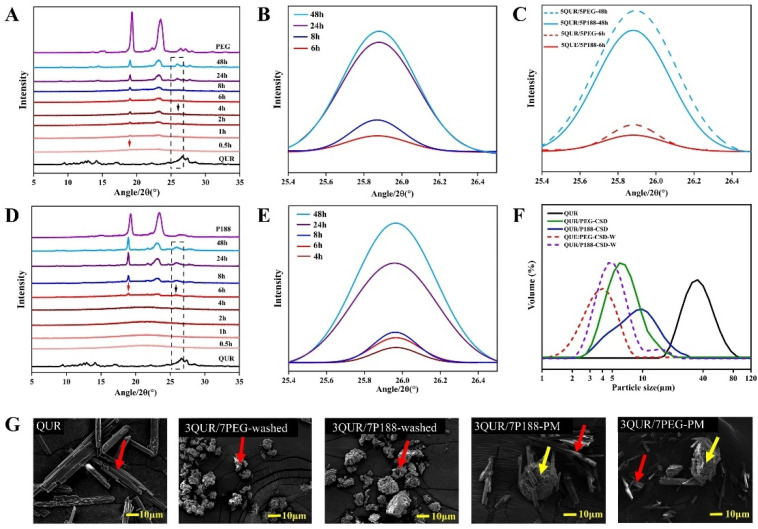
QUR crystalline size and crystallization kinetics. (**A**,**B**) QUR/PEG8000 crystallization kinetics; (**C**) Comparison of crystallization kinetics of QUR/P188-CSD and QUR/PEG8000-CSD at the same time point; (**D**,**E**) QUR/P188 crystallization kinetics; (**F**) Crystalline size of QUR and CSD; (**G**) SEM images of QUR and CSD.

In this study, the authors utilized PXRD to investigate the crystallization kinetics of QUR and polymers in CSD. As depicted in Figure 2A,B, with QUR/PEG8000-CSD, PEG8000 started crystallizing at 0.5 h, while QUR began to crystallize at 4 h. The diffraction peak intensity of QUR increased over time, indicating a gradual growth of QUR. Regarding QUR/P188-CSD (Figure 2D,E), both QUR and P188 were initially in an amorphous state at 0 h. Crystallization of both components began simultaneously at 6 h [24]. The crystal growth rate of QUR was faster in PEG8000 compared to P188, suggesting that the initial crystallization of PEG8000, due to intermolecular interactions and steric hindrance, better controlled the crystal growth of QUR [14,16]. The crystallinity of QUR in P188 was lower than that in PEG8000 (Figure 2C) [16].

QUR, P188, PEG8000, and CSDs were analyzed utilizing PSA and SEM to validate the crystalline size of QUR in CSD. The PSA test of the two CSDs demonstrated that the drug crystalline size of all CSDs was smaller than the API (Figure 2F). The crystalline size of QUR/PEG8000-CSD (Dv_(0.5)_ = 7.61 ± 0.20 μm) was smaller than the crystalline size of QUR/P188-CSD (Dv_(0.5)_ = 8.3 ± 1.29 μm). To further explore the crystalline size of QUR in CSD, it underwent rinsing to remove the water-soluble polymer in CSD, retaining the sample powder of QUR in CSD for testing. Compared to the unwashed CSD, the crystalline size of QUR in the washed CSD was significantly reduced, and the QUR crystalline size following washing in QUR/PEG8000-CSD (Dv_(0.5)_ = 5.35 ± 0.41 μm) was smaller than the crystalline size of QUR/P188-CSD (Dv_(0.5)_ = 6.72 ± 0.28 μm). This result mirrored the interaction results, i.e., the stronger the interaction, the smaller the drug crystalline size.

After rinsing with deionized water to remove polymer materials and vacuum drying to remove moisture, the surface morphology of the prepared CSD samples was also observed with SEM. The QUR surface exhibited a rod-like structure, while P188 had cluster-like features, and the CSD surface following rinsing was fluffy and rough (Figure 2G). Macroscopic comparison following rinsing demonstrated that the drug crystal crystalline size of QUR in both CSDs was smaller than the raw QUR, and the effect of PEG8000 on QUR crystalline size was more significant (Figure 2G). According to the Oswald ripening effect [14], the drug crystalline size was expected to increase during the washing away of polymer materials with deionized water. However, the crystalline sizes observed in CSD were still smaller than those of QUR. In summary, the crystal crystalline size trend of QUR was QUR>QUR/P188-CSD>QUR/PEG8000-CSD.

### 2.4. In Vitro Dissolution Rate

#### 2.4.1. Equilibrium Solubility of QUR

QUR exhibited extremely low solubility in pure PBS, with a solubility of 0.45 μg/mL at 25 °C (Table 2). The addition of different concentrations of polymers to the PBS solution significantly affected the solubility of QUR. Both P188 and PEG8000 acted as solubilizing agents for QUR. As the concentration of polymer increased, the solubilizing effect of P188 on QUR was more significant than that of PEG8000 (*p* < 0.05).

#### 2.4.2. Intrinsic Dissolution Rate (IDR)

IDR is a crucial metric for understanding how drug–polymer interactions influence the initial release of a drug from SD. Compared with other experiments, the specific surface area, pH, stirring speed, and ionic strength of the medium were unaltered in the IDR experiment, ensuring that the fixed release surface area mitigated the effects of drug aggregation and uneven crystalline sizes [25]. This study aimed to evaluate how polymers impact the drug release rate in a simulated gastric juice environment (pH 1.4), closely mirroring in vivo and in vitro dissolution rates [26].

**Figure 3:**
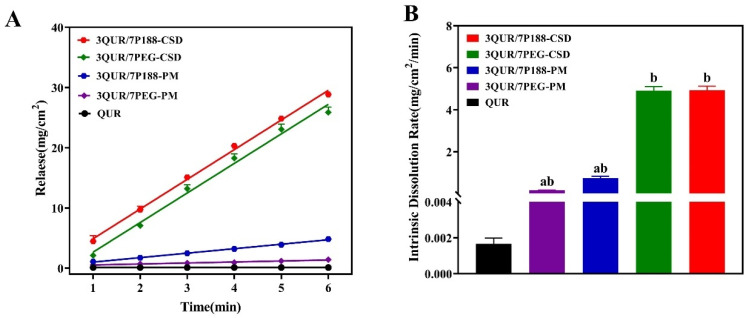
Intrinsic dissolution rates of QUR, QUR/P188 system and QUR/PEG8000 system at pH 1.4 (n = 3). ^a^ *p* < 0.01 when compared with CSD; ^b^ *p* < 0.01 when compared with QUR and PM. (**A**) The curve of intrinsic dissolution of drugs in CSD over time; (**B**) The intrinsic dissolution rate of the drug in CSD.

In comparison, QUR had the slowest IDR (0.0017 mg/cm^2^/min), whereas the IDR of QUR/P188-PM (0.7426 mg/cm^2^/min) and QUR/PEG8000-PM (0.1659 mg/cm^2^/min) were faster than QUR, showing 436-fold and 98-fold increases, respectively (Figure 3). As an amphiphilic surfactant, P188 can substantially improve the solubility and wettability of QUR, consistent with the findings of apparent solubility [15]. However, the dissolution rate of CSD was also modified due to the change in QUR crystalline size in CSD. The IDRs of QUR/P188-CSD and QUR/PEG8000-CSD were 4.9283 mg/cm^2^/min and 4.9092 mg/cm^2^/min, representing a 2899-fold and 2888-fold increase compared to QUR, respectively. Despite the crystalline size difference (QUR/PEG8000-CSD being smaller than QUR/P188-CSD), the final IDR results were similar due to the effective wetting and solubilizing properties of P188. Both P188 and PEG8000 significantly enhanced the dissolution rate of the QUR-CSD formulation, indicating their potential to improve the in vitro dissolution and oral absorption of QUR.

#### 2.4.3. Effect of Polymers on QUR’s Ability to Maintain Supersaturation

Thermodynamically unstable supersaturated drugs may maintain a certain degree of supersaturation, but the drug will recrystallize via hydration over time, losing its solubilization effect [27]. The saturation (S) of a drug can be calculated using Equation (6).

**Figure 4:**
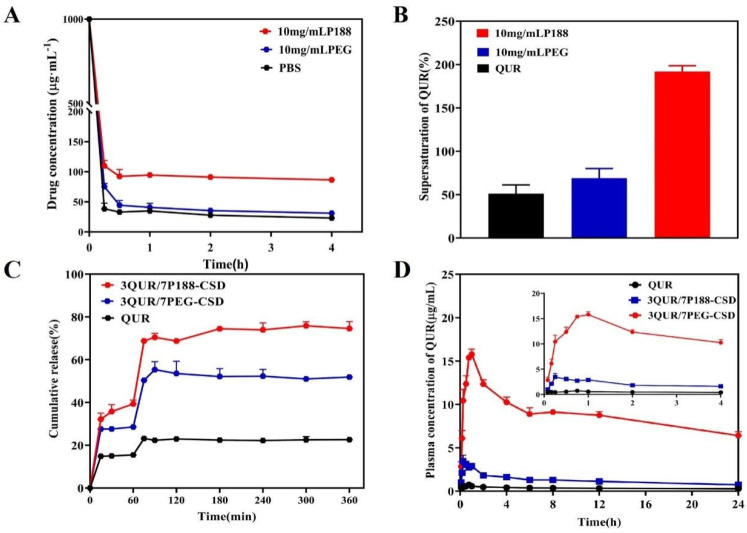
QUR dissolution rate in vivo and in vitro. Influence of polymers on QUR saturation in PBS solution. (**A**) Drug supersaturation kinetic curve in PBS solution (n = 3); (**B**) QUR supersaturation. (**C**) The pH conversion two-step dissolution of QUR (n = 3). (**D**) In vivo release behavior of QUR preparations (n = 6).

As depicted in Figure 4, the initial concentration of QUR was 1 mg/mL (approximately 2222 times the apparent solubility of QUR), dropping to 38 μg/mL within 20 min (Figure 4A). The *S* values of QUR after adding PEG8000 and P188 for 4 h were 69% and 191%, respectively (Figure 4B). The latter was much larger, indicating that PEG8000 could not maintain the supersaturation of QUR for a prolonged period [28]. In addition, P188’s ability to maintain QUR at high concentrations may be linked to its solubilization effect on QUR and its adsorption to the drug surface, controlling QUR aggregation and crystal growth [29].

#### 2.4.4. pH Conversion Two-Step Dissolution

The pH conversion two-step is designed to evaluate the dissolution rate of drugs in a gastrointestinal pH environment, which is crucial for predicting the in vivo drug release rate. Since the limited volume of gastric juice cannot completely dissolve insoluble drugs, conducting these studies under non-sink conditions better simulates drug release in gastrointestinal fluids.

As illustrated in Figure 4C, when the dissolution medium’s pH is 1.4, the drug releases quickly and reaches a saturated equilibrium state. Pure QUR achieved only 15% cumulative release in 1 h, whereas QUR/P188-CSD and QUR/PEG8000-CSD reached 39% and 28%, respectively. At pH 6.5, the drug dissolution of QUR in CSD increased. The cumulative dissolution rates at 6 h for QUR/P188-CSD and QUR/PEG8000-CSD were 75% and 52%, respectively, i.e., 3.4 times and 2.4 times higher than QUR (22%). Their cumulative dissolution areas under the curve (AUC) were 23,819.23 ± 491.46% and 17,172.90 ± 525.70%, respectively, i.e., 3 times and 2.3 times higher than QUR’s (7501.00 ± 153.10%). Additionally, the AUC of QUR/P188-CSD and QUR/PEG8000-CSD showed significant differences (*p* < 0.01).

These results indicate that although the stronger interaction between QUR and PEG8000 in CSD resulted in smaller drug crystalline size, the strong wetting and solubilizing effects of P188 on QUR, combined with its saturation maintenance effect, led to a superior dissolution rate for QUR/P188-CSD in the pH conversion two-step dissolution. This suggests that in the CSD system, factors such as the polymer’s wettability, the solubilizing effect of the polymer on the drug, and the ability to maintain supersaturation will impact the drug’s dissolution rate. Ultimately, the dissolution rate in the pH conversion two-step dissolution is the apparent result of considering all these factors.

### 2.5. Pharmacokinetics

The relative absorption of QUR/P188-CSD and QUR/PEG8000-CSD was enhanced compared to that of QUR (Figure 4D and Table 3). The AUCs_(0–24h)_ for QUR/P188-CSD and QUR/PEG8000-CSD were 29.73 ± 9.95 and 211.69 ± 22.48 μg/mL/h, respectively, representing increases of 3.5 and 25.0 times over QUR’s (8.46 ± 1.38 μg/mL/h). Additionally, the C_max_ for QUR/P188-CSD and QUR/PEG8000-CSD increased by 5.5 times and 22.6 times, respectively, compared to QUR. While the oral absorption of both CSDs improved markedly, there was not a strong correlation between the in vivo absorption and in vitro dissolution rates.

Generally, the in vitro dissolution rate of QUR/P188-CSD is superior to that of QUR/PEG8000-CSD, and its in vivo dissolution performance is also excellent. However, in this study, the results showed the opposite, suggesting that other factors influence drug absorption. A literature review indicated that both P188 and PEG8000 can alter intestinal permeability and enhance drug absorption, potentially related to P-gp activity [19,30]. Studies on ketoprofen and nadolol showed that P188 can increase drug permeability by inhibiting P-gp efflux and reducing membrane fluidity [30]. Conversely, PEG8000 affects P-gp activity and can enter cells via caveolin-mediated pathways [19]. We hypothesized that the significantly greater oral absorption of QUR/PEG8000-CSD compared to QUR/P188-CSD may have been due to PEG8000’s interaction with caveolin-mediated pathways. To test this hypothesis, we designed specific drug transmembrane transport experiments.

### 2.6. Cell Permeability

**Figure 5:**
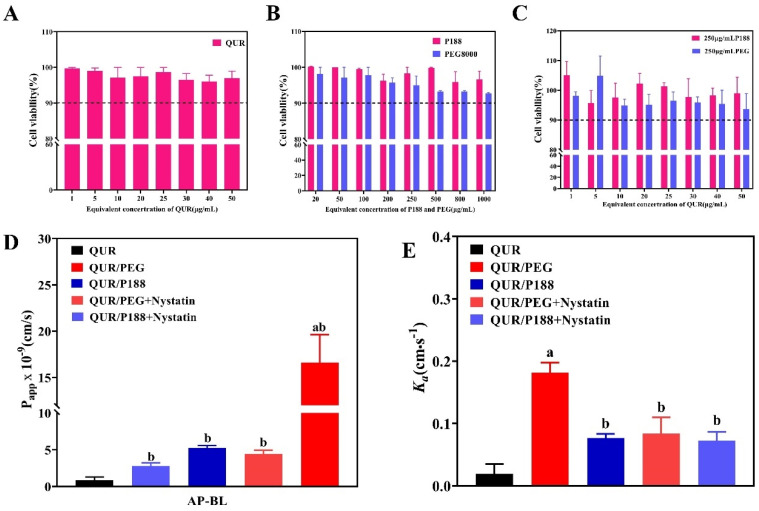
Transmembrane transport experiments of QUR in Caco-2 cells and single pass intestinal perfusion models (n = 6). (**A**) QUR cell activity test; (**B**) P188 and PEG8000 cell activity test; (**C**) QUR, P188, and PEG8000 synergistic cell activity tests; (**D**) QUR cell transmembrane experiment; (**E**) Absorption QUR, QUR/P188, and QUR/PEG8000 in single pass intestinal perfusion models. ^a^ *p* < 0.01 compared to QUR/PEG8000; ^b^ *p* < 0.01 when compared with QUR.

In this study, the CCK-8 method was used to assess the cytotoxicity of pure QUR, QUR/P188, and QUR/PEG8000 (Figure 5A). QUR exhibited low toxicity to Caco-2 cells at concentrations ranging from 1–50 μg/mL, maintaining cell viability above 95%. Thus, QUR is considered safe for experimental use within this concentration range. P188 and PEG8000 also demonstrated minimal cytotoxicity at increasing concentrations. However, PEG8000 showed significant toxicity at 0.5 mg/mL, reducing cell viability to below 95%, while P188 maintained cell survival rates above 95% at 1 mg/mL (Figure 5B). Consequently, for a synergistic toxicity evaluation, a polymer dose of 250 μg/mL was selected, and the drug concentration was set at 1–50 μg/mL. At these concentrations, cell viability remained above 95% (Figure 5C). Therefore, we used 25 μg/mL QUR and 250 μg/mL polymer for cell transmembrane experiments.

Caco-2 cell monolayers are a reliable model for mimicking human intestinal barriers, commonly used to predict in vivo permeability and absorption. Transport assays using Caco-2 cell monolayers help evaluate drug permeability and P-gp efflux transporters, the gold standard for inhibiting intestinal permeability [31]. We employed the Caco-2 monolayer model to compare the permeability effects of P188 and PEG8000 on QUR (Figure 5D). After culturing Caco-2 cells in 12-well Transwell culture plates for 21 days, the resistance value exceeded 200 Ω/cm^2^, and the Lucifer Yellow transmittance Papp was less than 1.0 × 10^−6^ cm·s^−1^, as required for permeability experiments. Additionally, the alkaline phosphatase activity on the AP side was substantially higher than on the BL side, indicating tight cell junctions and proper differentiation, making them suitable for transport experiments [32,33].

As illustrated in Figure 5D, the penetration rate of QUR Papp was the lowest, but this increased after treatment with P188 and PEG8000 [34]. However, the drug penetration rate in QUR/PEG8000-CSD was approximately 5 times higher than in QUR/P188-CSD. When a caveolin inhibitor (nystatin) was added [19], the penetration rate of QUR/P188-CSD remained unchanged (*p* > 0.05), while the penetration rate of the QUR/PEG8000-CSD significantly decreased (*p* < 0.01). These experiments confirmed that both P188 and PEG8000 enhanced the cell permeability and intestinal absorption of QUR. Furthermore, PEG8000 may have further increased the drug permeability and intestinal absorption through caveolin-mediated endocytosis.

### 2.7. In Vivo Single Pass Intestinal Perfusion

We conducted in vivo single pass intestinal perfusion experiments to further verify PEG8000 endocytosis mediated by caveolin. Both P188 and PEG8000 were found to enhance QUR absorption in the intestine (Figure 5E). The absorption rate constant (*K_a_*) of QUR/PEG8000-CSD was 0.1816 cm·s^−1^, which is 9.5 times and 2.4 times higher than those of QUR (*K_a_* = 0.0192 cm·s^−1^) and QUR/P188-CSD (*K_a_* = 0.0768 cm·s^−1^), respectively. The intestinal absorption of QUR/PEG8000-CSD was significantly reduced after the addition of caveolin inhibitors (*p* < 0.05).

In summary, both P188 and PEG8000 could enhance the cellular permeability and intestinal absorption of QUR. CSD prepared with PEG8000 as a carrier could be internalized into cells through caveolin-mediated endocytosis, leading to increased cell permeability and intestinal absorption. In contrast, CSD using P188 as a carrier did not utilize this transport mechanism. This difference may explain the higher absorption of QUR/PEG8000-CSD compared to QUR/P188-CSD.

## 3. Materials and Methods

### 3.1. Reagent and Materials

**Figure 6:**
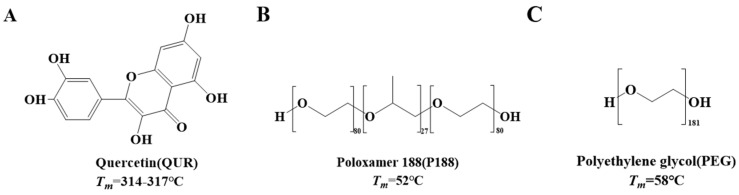
Structures of Quercetin (**A**), Poloxamer 188 (**B**), and Polyethylene glycol (**C**).

Quercetin (purity > 98%), apigenin (purity > 98%), the PEO-PPO-PEO triblock copolymer poloxamer 188 (purity > 98%), and polyethylene glycol 8000 (purity > 98%) were sourced from Beijing Coupling Technology Co., Ltd. (Beijing, China). High-Performance Liquid Chromatography (HPLC) grade methanol and analytical grade dichloromethane were obtained from Merck Co., Ltd. (Darmstadt, Germany). Buffer salts and other analytical-grade reagents were procured from Tianjin Damao Chemical Reagent Technology Co., Ltd. (Tianjin, China). The chemical structures of the model drugs and polymer materials used in the experiment are depicted in Figure 6. DMEM culture medium, trypsin, and fetal calf serum were purchased from Pronosai Life Technology Co., Ltd. (Wuhan, China). The CCK-8 detection kit was acquired from Elabscience Biotechnology Co., Ltd. (Wuhan, China). Reagents like nystatin and glucose were obtained from McLean Biochemical Technology Co., Ltd. (Shanghai, China). The human colon adenocarcinoma cell line Caco-2 cells were sourced from Sabikon Biotechnology Co., Ltd. (Shanghai, China).

### 3.2. Preparation of CSD and Physical Mixing (PM)

A mixture of QUR/P188 and QUR/PEG8000 (*w*/*w*, 30/70) was dissolved in dichloromethane at a total concentration of 5 wt%. This solution was then subjected to a rotary evaporator (N-1300 EYEL4, Shanghai, China) with operating parameters set to a rotation speed of 150 rpm and a water bath temperature of 40 °C. Following preparation, the resulting product was placed under vacuum at room temperature for at least 24 h to remove any residual solvent. The QUR/P188-CSD and QUR/PEG8000-CSD were dried under vacuum for at least 24 h and subsequently stored in a desiccator at 25 °C.

The physical mixtures of QUR with P188 and PEG8000 (QUR/P188-PM and QUR/PEG8000-PM) were prepared by sieving and mixing the powders of QUR and the two polymers to ensure uniform distribution.

### 3.3. Scanning Electron Microscopy (SEM)

The microstructure and surface morphology of the solid dispersion powder were examined using a SEM (Merlin Compact, Jena, Germany) at an excitation voltage of 10 kV. Samples were mounted on a copper platform and coated with gold for 180 s prior to observation.

### 3.4. Powders X-Ray Diffraction (PXRD)

The pure QUR, P188, QUR/P188-CSD, and QUR/PEG8000-CSD powders were characterized using a PXRD (ESCALAB™ XI+, Waltham, MA, USA) with a voltage of 40 kV and a current of 200 mA. The samples were scanned from 2θ = 5° to 35° at a scanning speed of 1°/min, with a step size of 0.01°.

### 3.5. Laser Particle Size Analyzer (PSA)

The crystalline size of the CSD was determined using a Mastersizer 2000 (Malvern Instruments, Malvern, UK) in dry test mode. Approximately 1.0 g of the sample was added to the dry test sample cell. The refractive index (RI) of the dispersion medium was set to 1.00, with the RI of QUR at 1.642 and an absorption index of 0.01. The sample detection duration was 10 s. The dispersion pressure was set at 2.0 bar, the injection speed at 50%, the slit width at 1.5 mm, and the shading range was 1.0 to 5.0%.

### 3.6. Hansen Solubility Parameters of Drug and Polymer

The partial solubility parameters were calculated using the Group Contribution Method (GCM). The Hansen solubility parameter method is the most commonly used approach for predicting drug–polymer miscibility. This method determines the compatibility between molecules based on calculated solubility parameters (Δδ_t_) by evaluating the difference between them [35]. The interaction parameters were calculated using Cohesive Energy Density (Equation (1)). The total solubility parameters of drugs and polymers were determined using Equation (2). GCM (Equation (3)) was employed to calculate the partial solubility parameters of a substance.

(1) $$\delta ={\left(\mathrm{CED}\right)}^{0.5}={(\Delta E_{V}/V_{m})}^{0.5}$$

(2) $$\delta_{t}=\delta_{d}^{2}+\delta_{p}^{2}+\delta_{h}^{2}$$

(3) $$\delta_{d}=\frac{\sum F_{d}}{V};\delta_{p}=\frac{\sqrt{\sum F_{p}^{2}}}{V};\delta_{h}=\sqrt{\frac{\sum E_{h}}{V}}$$

where ΔE_v_ represents the evaporation energy, V_m_ is the molar volume of the substance, δ_p_, δ_h_, and δ_d_ represent the solubility parameters of polarity, hydrogen bond, and dispersion, F_d_ represents the molar absorption constant of the dispersion group, F_p_ denotes the molar absorption constant of the polar group, E_h_ is the hydrogen bonding energy, and V represents the molar volume of the substance.

### 3.7. ^1^H-Nuclear Magnetic Resonance (^1^H-NMR)

To understand the molecular mechanisms of drug–polymer interaction, QUR, P188, and the different drug-loading CSDs were dissolved in deuterochloroform. Their 1H-NMR spectra were obtained at room temperature using an AVANCE NEO 600 (Bruker BioSpin GmbH, Rheinstetten, Germany). The deuterochloroform solvent signal was used as the reference (methyl sulfoxide, 77.160 ppm). Spectral assignments for QUR with P188 and PEG8000 were accomplished based on literature reports.

### 3.8. Determination of Interaction Parameter χ

Samples were analyzed using DSC with a slow scanning heating method [36]. The samples were heated at rates of 1, 2, 5, and 10 °C/min until completely melted. The melting temperature was determined at the intersection of the melting curve and baseline. The activity of QUR in P188 (α_d_) was calculated using the Van’t Hoff equation (Equation (4)), and the interaction parameters (χ value) between drugs and polymers were calculated using the Flory-Huggins equation (Equation (5)).

(4) $$\ln\alpha_{d}=\Delta H_{m}/R\left(1/T_{m}-1/T\right)$$

where Δ*H_m_* is the drug molar melting enthalpy, *Tm* represents the drug molecule melting temperature, *T* is the drug/polymer system equilibrium melting temperature, and *R* is the gas constant.

(5) $$\ln\alpha_{d}=\ln\Phi_{d}+\left(1-1/x\right)\Phi_{p}+\chi \Phi_{p}^{2}$$

where *Φ_d_* is the volume constant of the drug, *Φ_p_* is the polymer volume constant, *x* represents the drug/polymer molar volume ratio, and *χ* denotes the Drug–Polymer Interaction Parameters.

### 3.9. Dissolution Kinetics In Vitro

The concentration of QUR was determined using high-performance liquid chromatography (HPLC) (Agilent 1260 Series, Palo Alto, CA, USA) with UV detection at 374 nm. A Diamonsil C18 column (4.6 × 150 mm, 5 μm) was used, with a mobile phase of methanol/0.1% formic acid water (55/45, *v*/*v*) at a flow rate of 1.00 mL/min. The column temperature was maintained at 30 °C, and the injection volume was 10.00 μL. The HPLC method was validated for specificity, calibration curve, precision, repeatability, stability, and recovery.

To assess the apparent solubility, an excess amount of QUR was added to PBS buffer containing P188 and PEG8000 at different concentrations (0.3, 1, 3 mg·mL^−1^). The mixture was vortexed, sonicated for 30 min, and shaken at room temperature for 24 h. After centrifugation at 15,000 rpm for 15 min, the supernatant was diluted with methanol, and the QUR concentration was determined by HPLC.

To examine the intrinsic dissolution, 150 mg of pure QUR, PM, and CSDs were compressed separately into cylinder tablets with a cylindrical diameter of 8 mm. Each tablet was sealed in a syringe with paraffin wax, exposing only one surface to ensure a uniform dissolution area. A sample of 20 mL of 0.05 M HCl (pH 1.4) dissolution medium was placed in a 25 mL beaker. Samples of 0.3 mL were extracted every 0.5 min under magnetic stirring (400 rpm) and immediately centrifuged at 15,000 rpm for 3 min. The supernatant was analyzed for QUR concentration using HPLC.

pH conversion two-step dissolution of CSDs was then assessed. The dissolution profile of the formulations (tablets) was determined under non-sink conditions using a ZRS-6G dissolution apparatus (Tianjin Jingtuo Instrument Technology Co., Ltd., Tianjin, China). The dissolution medium was 300 mL of 0.05 M HCl (pH 1.4) containing 0.1% Tween 80, and the treatment lasted for 1 h. The pH was then adjusted to 6.5 by adding 450 mL of 0.1 M Na_2_HPO_4_ solution, and the test continued for an additional 4 h. Dissolution conditions included paddle stirring at 75 rpm and a temperature of 37 °C. Sampling time points were at 30, 60, 75, 90, 120, 180, 240, and 300 min. The samples were centrifuged at 15,000 rpm for 3 min, double diluted with methanol, and analyzed for QUR presence using HPLC/UV.

### 3.10. Effects of Different Polymers on Supersaturated QUR

To evaluate the ability of P188 and PEG8000 to maintain QUR supersaturation, we prepared a supersaturated QUR solution by dissolving QUR in dimethyl sulfoxide. A 100 μL sample of supersaturated drug solution was added to 10 mL of PBS containing different polymers (10 mg/mL), resulting in a 1 mg/mL QUR supersaturated solution. The mixture was incubated at 37 °C with shaking (100 rpm). Samples of 0.3 mL were extracted at 0.25, 0.5, 1, 2, and 4 h, centrifuged at 13,000 rpm for 5 min, and the supernatant was analyzed by HPLC/UV. The saturation (S) of a drug could be calculated using Equation (6).

(6) $$S=\frac{C}{C_{\mathrm{eq}}}$$

where *S* is the supersaturation of the drug, *C* represents the drug concentration in the solution, and *C_eq_* denotes the equilibrium solubility of the crystalline drug.

### 3.11. Pharmacokinetic Analysis

Sprague-Dawley (SD) rats (n = 6), weighing 180 ± 20 g, were used to evaluate in vivo pharmacokinetics. The rats were fasted overnight before administration and were fed 4 h post-administration. QUR, QUR/P188-CSD, and QUR/PEG8000-CSD were administered via gavage at a dose of 150 mg/kg. Blood samples (200–400 μL) were collected at 0.08, 0.17, 0.25, 0.50, 0.75, 1, 2, 4, 6, 8, 12, and 24 h after administration and centrifuged at 10,000 rpm for 10 min. The plasma was stored at −80 °C until analysis.

After thawing the plasma samples, 100 μL was taken and mixed with 25 μL of internal standard solution (14 μg/mL apigenin) and 100 μL of 25% hydrochloric acid. The mixture was vortexed and heated in a water bath at 90 °C for 15 min. Next, 175 μL of anhydrous ethanol was added, vortexed, and centrifuged (10,000 rpm, 10 min). The supernatant was collected, and the QUR content was determined using HPLC.

### 3.12. Caco-2 Cell Experiments

The cytotoxicity levels of QUR, QUR/P188-CSD, and QUR/PEG8000-CSD were assessed using the CCK-8 assay. Caco-2 cells were cultured in DMEM containing 10% (*v*/*v*) fetal calf serum, 100 units/mL penicillin, and 100 μg/mL streptomycin in a 5% CO_2_ incubator at 37 °C. Caco-2 cells in the logarithmic growth phase were digested with 0.25% trypsin, suspended in complete culture medium, and seeded in a 96-well culture plate at 5 × 10^4^ cells per well, with a volume of 100 μL per well. The cells were incubated for 24 h under standard conditions (37 °C, 5% CO_2_, and saturated humidity).

Pure QUR, P188, and PEG8000 were mixed at different concentrations using DMEM culture medium. The mixtures were then added to the 96-well plates containing the seeded cells and incubated for another 24 h. Afterward, 10 μL of CCK-8 reagent was added to each well. The absorbance at 460 nm was recorded using a microplate reader after incubation for 15, 30, and 60 min. The cell survival rates at different drug concentrations were calculated using Equation (7).

Note: The optimal incubation time was determined to be 30 min after adding the CCK-8 reagent. Incubating too long results in excessively high OD values, while shorter incubation times yield too low OD values.

(7) $$\mathrm{Survival}(\%)=\frac{\left({\mathrm{Sample}}_{P}-{\mathrm{Sample}}_{B}\right)}{\left({\mathrm{Sample}}_{N}-{\mathrm{Sample}}_{B}\right)}$$

Survival represents the cell survival rate; Sample_P_ is the OD value of the positive control group; Sample_N_ denotes the OD value of the negative control group; and Sample_B_ is the OD value of the blank control group.

To assess the cell permeability, 2 × 10^5^ Caco-2 cells were seeded in a 12-well Transwell culture plate. A 0.5 mL cell suspension was added to the apical (AP) side of the Transwell plate, and 1.5 mL of complete culture medium was added to the basal (BL) side. The plates were incubated at 37 °C, 5% CO_2_, and saturated humidity for 21 days, with the culture medium replaced every three days. The integrity of the Caco-2 monolayer was evaluated by measuring transepithelial electrical resistance (TEER) with a resistometer, assessing polarity with alkaline phosphatase activity, and determining transmittance using fluorescein. A TEER greater than 200 Ω/cm^2^, higher alkaline phosphatase activity on the AP side compared to the BL side, and fluorescin transmittance less than 1.0 × 10^−6^ cm·s^−1^ indicated a suitable monolayer for permeability studies.

Drug concentrations were determined based on cytotoxicity experiments. The drug was added to the AP side of the Transwell plate, while 1.5 mL of PBS was added to the BL side. The plate was incubated at 37 °C and 5% CO_2_. Samples were taken from the BL side at 30, 60, 90, and 120 min, centrifuged at 13,000 rpm for 5 min, and analyzed using LC-MS/MS to determine drug concentration. The transmembrane permeability *P_app_* was calculated using Equation (8).

(8) $$P_{\mathrm{app}}=(\mathrm{dQ}/\mathrm{dt})/(A\cdot C0)$$

where dQ/dt is the permeation rate per unit time; A represents the surface area of the polycarbonate membrane (1.12 cm^2^); and C0 is the initial concentration of the drug (μg/mL).

### 3.13. In Vivo Single Pass Intestinal Perfusion Experiment

SPF grade SD male rats, aged 6–8 weeks and weighing 180 ± 20 g, were purchased from Spefford Biotechnology Co., Ltd. (Beijing, China). The rats were separated into five groups: QUR, QUR/P188-CSD, QUR/PEG8000-CSD, QUR/P188-CSD plus caveolin inhibitor, and QUR/PEG8000-CSD plus caveolin inhibitor.

K-R nutrient solution was prepared by dissolving 7.8 g of NaCl, 1.37 g of NaHCO_3_, 0.35 g of KCl, 0.32 g of NaH_2_PO_4_, 0.02 g of MgCl_2_, 0.37 g of CaCl_2_, and 1.40 g of glucose in distilled water. CaCl_2_ was dissolved separately and added dropwise, while glucose was added just before use. The solution was diluted to 1 L with distilled water and stored at 4 °C. Drugs were precisely weighed and dissolved in the nutrient solution to create a perfusate with a concentration of 25 μg/mL.

Rats were fasted for 12 h before perfusion but had free access to water. Under continuous anesthesia with isoflurane (dosage according to body weight), a 3–4 cm incision was made along the midline of the abdomen. The common bile duct was ligated to prevent enterohepatic circulation. A 25 cm intestinal segment was identified and cannulated. Intestinal contents were flushed with 37 °C normal saline until clear. The remaining saline was removed with air, and the medicated perfusate was introduced at a flow rate of 0.2 mL/min for 30 min to equilibrate the intestine before timing. During perfusion, the rat’s abdominal cavity was covered with gauze moistened with 37 °C saline to maintain the health of the intestinal segment and the rat’s body temperature. The inlet was perfused with a test solution in a pre-weighed tube, while the outlet collected the perfusate in another pre-weighed tube. Samples were collected every 15 min using different test and collection tubes.

After the experiment, the supply and receiving tubes were weighed to calculate the mass differences. Collected samples were centrifuged at 12,000 rpm for 10 min, and the supernatant was analyzed via HPLC to determine drug concentration. The absorption constant *K_a_* of QUR in the intestinal segment was calculated using Equations (9) and (10).

(9) $$K_{a}=(1-C_{\mathrm{correct}}/C_{\mathrm{in}})\frac{Q}{\pi r^{2}l}$$

(10) $$C_{\mathrm{correct}}=C_{\mathrm{out}}\frac{Q_{\mathrm{out}}}{Q_{\mathrm{in}}}$$

where *K_a_* represents the absorption rate constant; C_in_ and C_out_ are the mass concentrations of the perfusate at the intestinal inlet and outlet (µg/mL), respectively; l and r are the length (cm) and cross-sectional radius (cm) of the perfused intestinal segment; Q denotes the perfusion speed (mL/min); and Q_in_ and Q_out_ are the mass of the test tube filled with perfusate and the mass of the test tube following perfusion (g), respectively.

### 3.14. Statistical Analysis

Statistical analysis was performed using SPSS 28.0 (IBM Inc., Armonk, NY, USA) software. Measurement data are presented as mean ± standard deviation. All data conformed to independence, normality and homogeneity of variance. Student’s *t*-test was used to determine the significance between the two groups. A *p* value < 0.05 was considered statistically significant.

## 4. Conclusions

The aim of this study was to improve the dissolution rate and oral absorption of QUR CSD. Using ^1^H-NMR and DSC, we demonstrated that QUR/PEG8000 exhibited stronger intermolecular hydrogen bonding interactions and confirmed through theoretical calculations and actual measurements that QUR and PEG8000 had better miscibility. This provides strong evidence for the reduction in QUR crystalline size. Beyond crystalline size, the dissolution rate of QUR was influenced by the wettability and solubilization effects of the polymer, as well as the polymer’s ability to maintain drug supersaturation.

The pH conversion two-step dissolution results indicated that QUR/P188-CSD had a better dissolution rate compared to QUR/PEG8000-CSD. Both QUR/PEG8000-CSD and QUR/P188-CSD enhanced the oral absorption of QUR, increasing it by 25 times and 3.5 times, respectively, relative to pure QUR. This result contradicts the in vitro dissolution experiments. Studies using the Caco-2 monolayer cell model and single pass intestinal perfusion model revealed that caveolin-mediated pathways are involved in the transmembrane transport of PEG8000, making QUR/PEG8000-CSD more permeable than QUR/P188-CSD. This likely explains the greater oral absorption of QUR/PEG8000-CSD.

In summary, we have successfully developed QUR-CSD using P188 and PEG8000 as carriers, both of which effectively improved the drug dissolution rate and oral absorption. This work lays a theoretical foundation for the development of new oral QUR preparations.

## Figures and Tables

**Table 1:** Solubility parameters of QUR, P188, and PEG8000.

Drug/Polymer	δ_d_/MPa^0.5^	δ_p_/MPa^0.5^	δ_h_/MPa^0.5^	δ_t_/MPa^0.5^	Δδ_t_ (D, P)
QUR	22.37	6.85	1.1	23.42	N/A
P188	20.78	0.77	0.1	20.8	2.62
PEG8000	21.03	0.83	0.1	21.05	2.37

**Table 2:** Apparent solubility of QUR in various polymers (n = 3).

Drug	Concentration (μg/mL)
QUR	0.45 ± 0.05
P188 (0.3 mg/mL)	0.56 ± 0.08
P188 (1 mg/mL)	0.97 ± 0.05
P188 (3 mg/mL)	1.63 ± 0.03
PEG8000 (0.3 mg/mL)	0.51 ± 0.05
PEG8000 (1 mg/mL)	0.86 ± 0.03
PEG8000 (3 mg/mL)	0.90 ± 0.07

**Table 3:** Pharmacokinetic parameters (n = 6).

Parameters	QUR	QUR/P188-CSD	QUR/PEG8000-CSD
AUC_(0–24)_ (μg/mL/h)	8.46 ± 1.38	29.73 ± 9.95	211.69 ± 22.48
AUC_(0–∞)_ (μg/mL/h)	19.08 ± 7.51	74.85 ± 80.42	563.09 ± 267.75
C_max_ (μg/mL)	0.71 ± 0.19	3.89 ± 1.65	16.03 ± 0.95
T_max_ (h)	0.90 ± 0.60	0.40 ± 0.22	0.92 ± 0.13
t_1/2_ (h)	28.46 ± 20.83	27.67 ± 24.19	35.84 ± 20.05
CL_z/F_ (L/h/kg)	9.00 ± 3.67	3.33 ± 1.73	0.30 ± 0.10
MRT_(0–24)_ (h)	10.94 ± 1.37	9.16 ± 1.03	10.48 ± 0.36
MRT_(0–∞)_ (h)	46.73 ± 28.62	36.86 ± 35.74	51.05 ± 29.18

## Data Availability

The datasets used or analyzed during the current study are available from the corresponding author upon reasonable request.
